# Comparative mammary oncology: canine model

**DOI:** 10.1186/1753-6561-7-S2-K6

**Published:** 2013-04-04

**Authors:** Geovanni Dantas Cassali

**Affiliations:** 1 Laboratório de Patologia Comparada – Departamento de Patologia Geral – ICB/UFMG, Brazil

## 

Mammary tumors are the most frequent neoplastic processes of the female dog and represent a problem of large impact in veterinary medicine. In this context, many efforts are being directed towards adopting criteria towards standardization of the diagnosis, understanding behavior and tumor evolution and evaluating prognostic and predictive factors such as: morphology, oncogenes expression and genetic alterations. The knowledge and adoption of these parameters are fundamentally important for choice and success of therapies that promote reduction of tumoral recurrence and increase in global survival. In addition, mammary tumors of female dog present epidemiological, clinical, biological and genetic similarities with mammary neoplasms in women and are therefore considered important models in comparative studies. The aim of our group is to evaluate clinical, morphological and molecular aspects of canine mammary tumors in order to identify comparative aspects with human breast cancer. Canine mammary tumors using TNM staging, histological grade, mitotic count and veterinary and human classifications were compared and reviewed. Results demonstrate that mammary tumor types in human and dogs are similar, although the relative frequency of each tumor type is different. Using immunohistochemical methodology, was standardized a series of specific human antigen antibodies in canine tissue. The antibodies were selected based on their ability to classify tumor types and to provide prognostic and predictive information previously described in human breast cancer studies. Results show that the expression of most markers is similar in benign and malignant mammary tumors in both species. It was also evaluated the prognostic value of mitotic index and histological malignancy grade demonstrating a sound correlation between those methods and the prognostic factors like in human breast cancer studies. Studies with found the Her-2 expression to be positively associated with nuclear pleomorphism, histological grade and mitotic count. Positive association was found between nuclear pleomorphism and MIB-1 index. These results imply that some tumor biological and morphological characteristics are associated with canine mammary gland tumors as it has been seen in human breast cancer. Studying benign non-neoplastic lesions was verified that they are pathologically and immunophenotypically similar to those in the human breast. In our diagnostic routine, mixed tumours are the most common tumour types in the female canine mammary gland. These tumours exhibit a complex histological pattern due to the presence of epithelial and mesenchymal elements and have the capacity to undergo malignant transformation, resulting in carcinomas. The origin of the several mixed tumour components is a subject of a long-standing controversy and is not yet fully understood. A suggested hypothesis states that these components originate from stem cells with a high divergence capability. This assumption is grounded on immunohistochemical studies and on the observation that the epithelial and mesenchymal components of mixed tumours are monoclonal. Carcinomas in benign mixed tumors (CBMT) are the most common malignant tumor in female dogs and may serve as a model for studies on tumor progression. Versican expression in *in situ* and invasive carcinomatous areas of CBMT was evaluated, verifying possible associations with other classic prognostic factors and overall survival. Results suggest that, as the myoepithelium gains mesenchymal characteristics, a decrease in p63 and α-SMA molecule expression and increase in versican expression occurs. In addition, the direct relation between versican and invasion suggests the role of this molecule in tumor progression. In CBMT were also investigated morphological aspects and their immunophenotypical profiles, through an immunohistochemical panel based on five molecular markers (ER, PR, HER2, CK5 and EGFR). It was concluded that CBMT are predominantly characterized as low-grade malignancy neoplasms. The various immunophenotypic profiles suggest the origin of these lesions in more than one cell type (luminal and myoepithelial). Invasive micropapillary carcinoma (IMPCa) of the mammary gland was first described by our group and should be highlighted. Despite its rare occurrence in humans and dogs, it has a distinct aggressive behaviour and poor prognosis. Were evaluated clinicopathological and immunophenotypical characteristics as well as the overall survival of canine IMPCa. Findings demonstrate that canine IMPCas are similar to human IMPCas, presenting aggressive behaviour with high rates of metastasis to regional lymph nodes and short overall survival and should be considered important lesions of the mammary gland in dogs. The distribution and intensity of lymphocytic responses in canine mammary tumors, in particular the relative abundance of the lymphocyte subpopulations which define whether the inflammatory process will act as a promoter or inhibitor of tumor development and metastasis were analyzed. Morphologic and immunohistochemical studies should be related with clinical aspects and serve as prognostics and predictive markers. Then patients suffering from mammary tumors with advanced clinical staging were studied. Analyzing the overall survival of animals treated only with surgery compared to those complementarily treated with carboplatin and Cox-2 inhibitors, the benefit of these patients from complementary therapy was verified. Carboplatin as an only drug was indicated for treating mammary tumors of female dogs in advanced clinical staging, with minimal side effects and easy administration. Limited therapeutic resources in mammary neoplasms of the female dog, the confirmed benefits of tamoxifen in human mammary tumors and insufficient data available on the canine specie justified a study that addressed additional effects of this drug in veterinary medicine. Side effects of oral administration of tamoxifen in spayed and intact female dogs were studied and were verified similar side effects to those observed in women with breast cancer treated with this medication. It was investigated the potential prognostic value of serum tumors markers and increased CA15.3 and LDH serum levels were directly related to the presence of regional lymph node and distant metastases. CEA showed no significant change among the groups (healthy, non-metastatic and metastatic mammary cancer groups). Thus, we suggest the possibility of using the criteria and the more extensive experience of human studies for the diagnosis and, in the future, treatment, of canine mammary gland tumors. On the other hand, these results reinforce the theory that spontaneous canine mammary tumors may be used as models for studies evaluating the mechanisms involved in mammary gland carcinogenesis, development of novel cancer therapeutics and may be relevant for human breast cancer studies.

## Competing interests

There are no competing interests in this presentation.

